# Supplementary material for: The Causal Relationship Between Dietary Factors and the Risk of Intracranial Aneurysms: A Mendelian Randomization Analysis
Source: Biomedicines. 2025 Feb 20;13(3):533. doi: 10.3390/biomedicines13030533 (PMC11940589; doi:10.3390/biomedicines13030533)

The Causal Relationship Between Dietary Factors and the Risk of Intracranial Aneurysms: A Mendelian Randomization Analysis

Longyuan Li †, Jiaxuan Li †, Mei Chang, Xin Wu, Ziqian Yin, Zhouqing Chen * and Zhong Wang *

Department of Neurosurgery & Brain and Nerve Research Laboratory, The First Affiliated Hospital of Soochow University, Suzhou 215006, China
* Correspondence: zqchen6@163.com (Z.C.); wangzhong761@163.com (Z.W.)
† These authors contributed equally to this work.

Figure legends

Table S1: The results of Mendelian randomization analyses.

Figure S1: MR analysis for diet-related exposure factors on intracranial aneurysms (ebi-a-GCST90018815).

Figure S2: MR analysis for diet-related exposure factors on intracranial aneurysms (finn-b-I9_ANEURYSM).

Figure S3: Leave-one-out plot for the effect of fresh fruit intake on intracranial aneurysms (ebi-a-GCST90018815).

Figure S4: Leave-one-out plot for the effect of fresh fruit intake on intracranial aneurysms (finn-b-I9_ANEURYSM).

Figure S5: Leave-one-out plot for the effect of lamb/mutton intake on intracranial aneurysms (ebi-a-GCST90018815).

Figure S6: Leave-one-out plot for the effect of lamb/mutton intake on intracranial aneurysms (finn-b-I9_ANEURYSM).

Figure S7: Scatter plot for the effect of fresh fruit intake on intracranial aneurysms (ebi-a-GCST90018815).

Figure S8: Scatter plot for the effect of fresh fruit intake on intracranial aneurysms (finn-b-I9_ANEURYSM).

Figure S9: Scatter plot for the effect of lamb/mutton intake on intracranial aneurysms (ebi-a-GCST90018815).

Figure S10: Scatter plot for the effect of lamb/mutton intake on intracranial aneurysms (finn-b-I9_ANEURYSM).

Figure S11: Funnel plot for the effect of fresh fruit intake on intracranial aneurysms (ebi-a-GCST90018815).

Figure S12: Funnel plot for the effect of fresh fruit intake on intracranial aneurysms (finn-b-I9_ANEURYSM).

Figure S13: Funnel plot for the effect of lamb/mutton intake on intracranial aneurysms (ebi-a-GCST90018815).

Figure S14: Funnel plot for the effect of lamb/mutton intake on intracranial aneurysms (finn-b-I9_ANEURYSM).

Figure S15: Forest plot for the effect of fresh fruit intake on intracranial aneurysms (ebi-a-GCST90018815).

Figure S16: Forest plot for the effect of fresh fruit intake on intracranial aneurysms (finn-b-I9_ANEURYSM).

Figure S17: Forest plot for the effect of lamb/mutton intake on intracranial aneurysms (ebi-a-GCST90018815).

Figure S18: Forest plot for the effect of lamb/mutton intake on intracranial aneurysms (finn-b-I9_ANEURYSM).

Table S1: The results of Mendelian randomization analyses

Exposure	Outcome	SNPs	Adjust P	Cochran's Q		Pleiotropy			MR-PRESSO[a]			
				Q	P-value	MR-Egger intercept	SE	P-value	casual estimate	sd	P-value	Outliers
Alcohol intake frequency ukb-b-5779	finn-b-I9_ANEURYSM	95	0.96	102	0.269	-0.0196	0.0179	0.276	0.272	0.233	0.246	NA
	ebi-a-GCST90018815	96	1	93.6	0.521	0.0047	0.00442	0.29	0.0788	0.087	0.367	NA
Alcoholic drinks per week ieu-b-73	finn-b-I9_ANEURYSM	34	1	18.8	0.978	-0.0176	0.0223	0.436	-0.675	0.43	0.126	NA
	ebi-a-GCST90018815	34	0.91	32.6	0.486	0.00821	0.00658	0.221	0.0556	0.149	0.712	NA
Beef intake ukb-b-2862	finn-b-I9_ANEURYSM	14	1	8.09	0.838	0.039	0.0859	0.658	0.367	1.01	0.721	NA
	ebi-a-GCST90018815	14	0.88	12.6	0.476	0.0138	0.0628	0.829	-0.706	0.677	0.313	NA
Bread intake ukb-b-11348	finn-b-I9_ANEURYSM	27	0.98	29	0.31	0.0154	0.0502	0.762	0.0706	0.687	0.919	NA
	ebi-a-GCST90018815	30	0.86	31.7	0.332	-0.0444	0.0246	0.0814	0.0962	0.356	0.789	NA
Cereal intake ukb-b-15926	finn-b-I9_ANEURYSM	39	0.97	41.2	0.332	0.00343	0.0388	0.93	0.145	0.581	0.804	NA
	ebi-a-GCST90018815	38	0.58	29.1	0.82	-0.032	0.023	0.173	-0.358	0.286	0.219	NA
Cheese intake ukb-b-1489	finn-b-I9_ANEURYSM	61	1	62.9	0.373	-0.047	0.0303	0.127	-0.603	0.423	0.159	NA
	ebi-a-GCST90018815	61	1	75.6	0.084	-0.0129	0.0185	0.487	0.116	0.268	0.666	NA
Coffee intake ukb-b-5237	finn-b-I9_ANEURYSM	38	0.94	31	0.747	-0.0248	0.0179	0.174	-0.169	0.485	0.729	NA

	ebi-a-GCST90018815	38	0.96	45.7	0.154	0.00958	0.012	0.43	0.146	0.34	0.67	NA
Cooked vegetable intake ukb-b-8089	finn-b-I9_ANEURYSM	17	0.93	18.6	0.291	0.0263	0.148	0.861	-0.451	1.27	0.727	NA
	ebi-a-GCST90018815	17	0.94	14.7	0.55	-0.0806	0.0728	0.286	-0.416	0.625	0.515	NA
Dried fruit intake ukb-b-16576	finn-b-I9_ANEURYSM	41	0.92	31	0.845	-0.0383	0.0359	0.292	0.372	0.604	0.541	NA
	ebi-a-GCST90018815	40	1	44.3	0.259	0.00388	0.0239	0.872	-0.297	0.372	0.428	NA
Fresh fruit intake ukb-b-3881	finn-b-I9_ANEURYSM	53	0.93	38.7	0.914	-0.00404	0.0242	0.868	-0.268	0.64	0.678	NA
	ebi-a-GCST90018815	53	0.0059	53.1	0.433	-0.00934	0.0141	0.511	-1.42	0.439	0.0021	NA
Lamb/mutton intake ukb-b-14179	finn-b-I9_ANEURYSM	31	1	34.1	0.278	0.0384	0.0455	0.405	-0.517	0.974	0.599	NA
	ebi-a-GCST90018815	31	0.39	22.9	0.821	0.0134	0.0239	0.58	0.964	0.443	0.0373	NA
Non-oily fish intake ukb-b-17627	finn-b-I9_ANEURYSM	11	1	7.95	0.633	0.0961	0.0759	0.237	-1.74	1.17	0.167	NA
	ebi-a-GCST90018815	11	1	7.15	0.711	0.0564	0.0503	0.291	-0.327	0.661	0.631	NA
Oily fish intake ukb-b-2209	finn-b-I9_ANEURYSM	61	1	61.1	0.435	0.0172	0.0293	0.561	-0.335	0.467	0.476	NA
	ebi-a-GCST90018815	61	1	50.8	0.795	0.00839	0.0166	0.614	-0.163	0.248	0.513	NA
Pork intake ukb-b-5640	finn-b-I9_ANEURYSM	13	1	10.6	0.566	0.0584	0.0889	0.525	0.0386	1.31	0.977	NA
	ebi-a-GCST90018815	14	0.66	22.5	0.0484	0.00767	0.0573	0.896	1.48	0.923	0.134	NA
Poultry intake ukb-b-8006	finn-b-I9_ANEURYSM	7	1	4.71	0.581	-0.275	0.565	0.647	-1.2	1.32	0.393	NA

	ebi-a-GCST90018815	7	0.95	6.5	0.369	-0.133	0.31	0.685	-0.324	0.914	0.733	NA
Processed meat intake ukb-b-6324	finn-b-I9_ANEURYSM	23	0.97	18.7	0.661	0.0341	0.0559	0.549	0.133	0.692	0.849	NA
	ebi-a-GCST90018815	23	1	13.9	0.906	0.0356	0.041	0.395	0.303	0.36	0.409	NA
Salad / raw vegetable intake ukb-b-1996	finn-b-I9_ANEURYSM	19	1	16.9	0.533	-0.0715	0.0652	0.288	-0.579	1.03	0.58	NA
	ebi-a-GCST90018815	19	0.87	14	0.727	-0.0687	0.0386	0.0926	-0.686	0.641	0.296	NA
Tea intake ukb-b-6066	finn-b-I9_ANEURYSM	40	1	58.5	0.0229	-0.0103	0.0228	0.654	0.858	0.537	0.118	NA
	ebi-a-GCST90018815	39	0.89	34.2	0.646	0.00834	0.0118	0.485	0.0731	0.231	0.754	NA

SNPs, Single-nucleotide polymorphisms; NA: Not available;

a: The results of MR-PRESSO are from raw data.

Figure S1: MR analysis for diet-related exposure factors on intracranial aneurysms (ebi-a-GCST90018815).

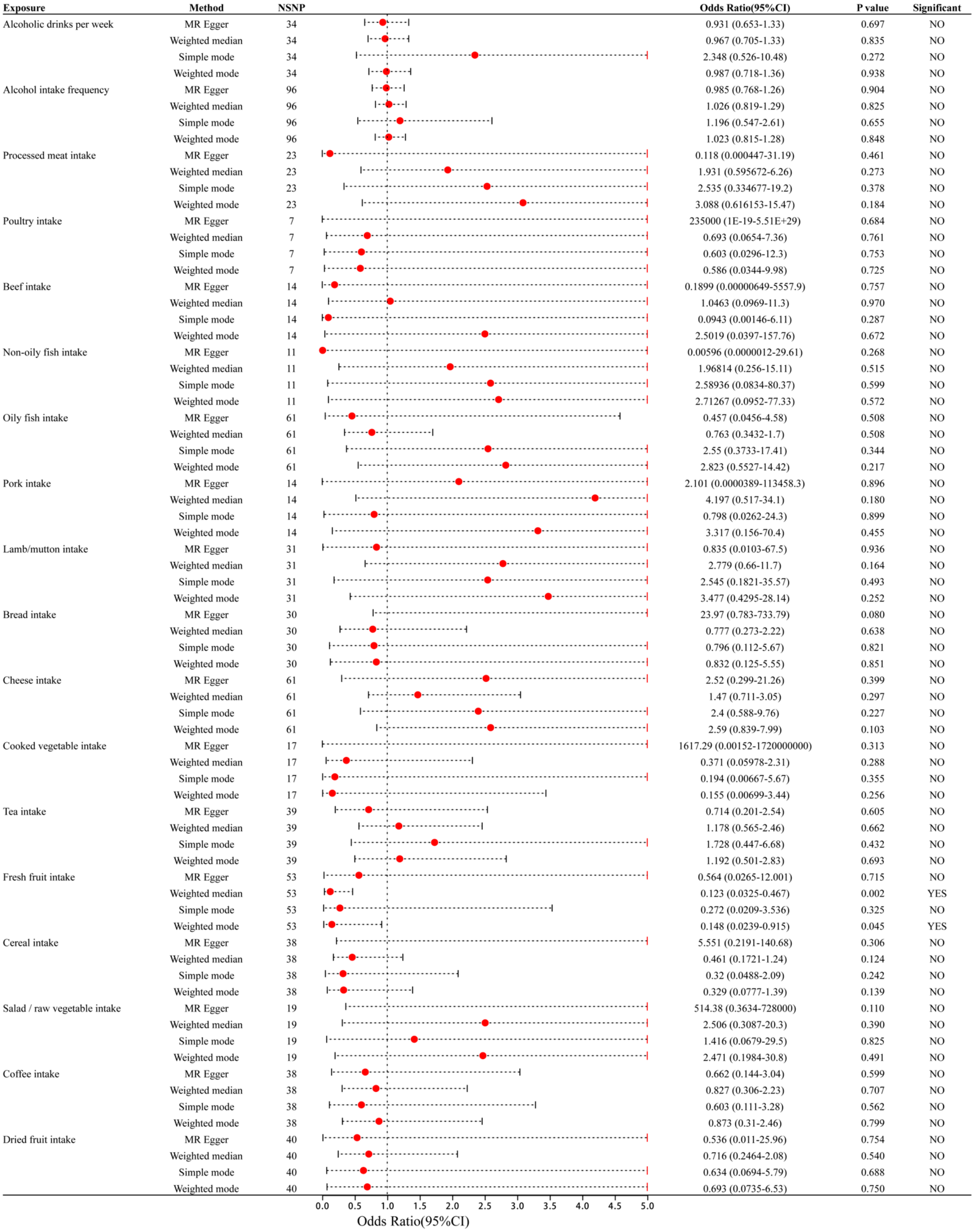

Exposure	Method	NSNP	Odds Ratio(95%CI)	P value	Significant
Alcoholic drinks per week	MR Egger	34	0.931 (0.653-1.33)	0.697	NO
	Weighted median	34	0.967 (0.705-1.33)	0.835	NO
	Simple mode	34	2.348 (0.526-10.48)	0.272	NO
	Weighted mode	34	0.987 (0.718-1.36)	0.938	NO
Alcohol intake frequency	MR Egger	96	0.985 (0.768-1.26)	0.904	NO
	Weighted median	96	1.026 (0.819-1.29)	0.825	NO
	Simple mode	96	1.196 (0.547-2.61)	0.655	NO
	Weighted mode	96	1.023 (0.815-1.28)	0.848	NO
Processed meat intake	MR Egger	23	0.118 (0.000447-31.19)	0.461	NO
	Weighted median	23	1.931 (0.595672-6.26)	0.273	NO
	Simple mode	23	2.535 (0.334677-19.2)	0.378	NO
	Weighted mode	23	3.088 (0.616153-15.47)	0.184	NO
Poultry intake	MR Egger	7	235000 (1E-19-5.51E+29)	0.684	NO
	Weighted median	7	0.693 (0.0654-7.36)	0.761	NO
	Simple mode	7	0.603 (0.0296-12.3)	0.753	NO
	Weighted mode	7	0.586 (0.0344-9.98)	0.725	NO
Beef intake	MR Egger	14	0.1899 (0.00000649-5557.9)	0.757	NO
	Weighted median	14	1.0463 (0.0969-11.3)	0.970	NO
	Simple mode	14	0.0943 (0.00146-6.11)	0.287	NO
	Weighted mode	14	2.5019 (0.0397-157.76)	0.672	NO
Non-oily fish intake	MR Egger	11	0.00596 (0.0000012-29.61)	0.268	NO
	Weighted median	11	1.96814 (0.256-15.11)	0.515	NO
	Simple mode	11	2.58936 (0.0834-80.37)	0.599	NO
	Weighted mode	11	2.71267 (0.0952-77.33)	0.572	NO
Oily fish intake	MR Egger	61	0.457 (0.0456-4.58)	0.508	NO
	Weighted median	61	0.763 (0.3432-1.7)	0.508	NO
	Simple mode	61	2.55 (0.3733-17.41)	0.344	NO
	Weighted mode	61	2.823 (0.5527-14.42)	0.217	NO
Pork intake	MR Egger	14	2.101 (0.0000389-113458.3)	0.896	NO
	Weighted median	14	4.197 (0.517-34.1)	0.180	NO
	Simple mode	14	0.798 (0.0262-24.3)	0.899	NO
	Weighted mode	14	3.317 (0.156-70.4)	0.455	NO
Lamb/mutton intake	MR Egger	31	0.835 (0.0103-67.5)	0.936	NO
	Weighted median	31	2.779 (0.66-11.7)	0.164	NO
	Simple mode	31	2.545 (0.1821-35.57)	0.493	NO
	Weighted mode	31	3.477 (0.4295-28.14)	0.252	NO
Bread intake	MR Egger	30	23.97 (0.783-733.79)	0.080	NO
	Weighted median	30	0.777 (0.273-2.22)	0.638	NO
	Simple mode	30	0.796 (0.112-5.67)	0.821	NO
	Weighted mode	30	0.832 (0.125-5.55)	0.851	NO
Cheese intake	MR Egger	61	2.52 (0.299-21.26)	0.399	NO
	Weighted median	61	1.47 (0.711-3.05)	0.297	NO
	Simple mode	61	2.4 (0.588-9.76)	0.227	NO
	Weighted mode	61	2.59 (0.839-7.99)	0.103	NO
Cooked vegetable intake	MR Egger	17	1617.29 (0.00152-1720000000)	0.313	NO
	Weighted median	17	0.371 (0.05978-2.31)	0.288	NO
	Simple mode	17	0.194 (0.00667-5.67)	0.355	NO
	Weighted mode	17	0.155 (0.00699-3.44)	0.256	NO
Tea intake	MR Egger	39	0.714 (0.201-2.54)	0.605	NO
	Weighted median	39	1.178 (0.565-2.46)	0.662	NO
	Simple mode	39	1.728 (0.447-6.68)	0.432	NO
	Weighted mode	39	1.192 (0.501-2.83)	0.693	NO
Fresh fruit intake	MR Egger	53	0.564 (0.0265-12.001)	0.715	NO
	Weighted median	53	0.123 (0.0325-0.467)	0.002	YES
	Simple mode	53	0.272 (0.0209-3.536)	0.325	NO
	Weighted mode	53	0.148 (0.0239-0.915)	0.045	YES
Cereal intake	MR Egger	38	5.551 (0.2191-140.68)	0.306	NO
	Weighted median	38	0.461 (0.1721-1.24)	0.124	NO
	Simple mode	38	0.32 (0.0488-2.09)	0.242	NO
	Weighted mode	38	0.329 (0.0777-1.39)	0.139	NO
Salad / raw vegetable intake	MR Egger	19	514.38 (0.3634-728000)	0.110	NO
	Weighted median	19	2.506 (0.3087-20.3)	0.390	NO
	Simple mode	19	1.416 (0.0679-29.5)	0.825	NO
	Weighted mode	19	2.471 (0.1984-30.8)	0.491	NO
Coffee intake	MR Egger	38	0.662 (0.144-3.04)	0.599	NO
	Weighted median	38	0.827 (0.306-2.23)	0.707	NO
	Simple mode	38	0.603 (0.111-3.28)	0.562	NO
	Weighted mode	38	0.873 (0.31-2.46)	0.799	NO
Dried fruit intake	MR Egger	40	0.536 (0.011-25.96)	0.754	NO
	Weighted median	40	0.716 (0.2464-2.08)	0.540	NO
	Simple mode	40	0.634 (0.0694-5.79)	0.688	NO
	Weighted mode	40	0.693 (0.0735-6.53)	0.750	NO

Figure S2: MR analysis for diet-related exposure factors on intracranial aneurysms (finn-b-I9_ANEURYSM).

Exposure	Method	NSNP	Odds Ratio(95%CI)	P value	Significant
Alcoholic drinks per week	MR Egger	34	1.309 (0.0972-17.62)	0.84	NO
	Weighted median	34	0.382 (0.0758-1.92)	0.243	NO
	Simple mode	34	0.483 (0.0239-9.76)	0.639	NO
	Weighted mode	34	0.335 (0.0286-3.92)	0.389	NO
Alcohol intake frequency	MR Egger	95	2.868 (0.651-12.63)	0.167	NO
	Weighted median	95	1.392 (0.707-2.74)	0.339	NO
	Simple mode	95	0.973 (0.209-4.52)	0.972	NO
	Weighted mode	95	1.43 (0.455-4.5)	0.542	NO
Processed meat intake	MR Egger	23	0.121 (0.0000763-191.97)	0.58	NO
	Weighted median	23	0.69 (0.091-5.23)	0.719	NO
	Simple mode	23	1.082 (0.0181-64.88)	0.97	NO
	Weighted mode	23	0.358 (0.00673-19.08)	0.618	NO
Poultry intake	MR Egger	7	31800000000 (1.1E-34-9.21E+54)	0.663	NO
	Weighted median	7	0.104 (0.00103-10.5)	0.336	NO
	Simple mode	7	0.0985 (0.000106-91.6)	0.531	NO
	Weighted mode	7	0.0985 (0.000158-61.6)	0.507	NO
Beef intake	MR Egger	14	0.0274 (0.0000000398-18906.74)	0.61	NO
	Weighted median	14	0.4503 (0.0245-8.26)	0.591	NO
	Simple mode	14	0.2598 (0.00285-23.63)	0.568	NO
	Weighted mode	14	0.4029 (0.00465-34.93)	0.696	NO
Non-oily fish intake	MR Egger	11	0.000076 (0.000000000361-15.97)	0.164	NO
	Weighted median	11	0.092308 (0.00302-2.83)	0.172	NO
	Simple mode	11	0.075293 (0.00031-18.27)	0.378	NO
	Weighted mode	11	0.093654 (0.00075-11.7)	0.359	NO
Oily fish intake	MR Egger	61	0.231 (0.00429-12.47)	0.474	NO
	Weighted median	61	0.476 (0.1294-1.75)	0.265	NO
	Simple mode	61	0.226 (0.01234-4.15)	0.321	NO
	Weighted mode	61	0.194 (0.01345-2.79)	0.233	NO
Pork intake	MR Egger	13	0.00378 (0.000000000156-91278.7)	0.533	NO
	Weighted median	13	1.1206 (0.0291-43.2)	0.951	NO
	Simple mode	13	0.63954 (0.000761-537.8)	0.899	NO
	Weighted mode	13	0.49292 (0.00122-199.8)	0.821	NO
Lamb/mutton intake	MR Egger	31	0.0153 (0.00000393-59.39)	0.33	NO
	Weighted median	31	0.7291 (0.0561-9.47)	0.809	NO
	Simple mode	31	1.2014 (0.00533-271)	0.948	NO
	Weighted mode	31	0.7819 (0.00807-75.77)	0.917	NO
Bread intake	MR Egger	27	0.35 (0.000321-381.14)	0.771	NO
	Weighted median	27	2.1 (0.296813-14.91)	0.457	NO
	Simple mode	27	2.53 (0.070104-91.12)	0.616	NO
	Weighted mode	27	4.09 (0.229108-73.08)	0.347	NO
Cheese intake	MR Egger	61	8.291 (0.23662-290.53)	0.248	NO
	Weighted median	61	0.403 (0.11826-1.37)	0.146	NO
	Simple mode	61	0.129 (0.00648-2.58)	0.185	NO
	Weighted mode	61	0.148 (0.00573-3.83)	0.255	NO
Cooked vegetable intake	MR Egger	17	0.05 (3.040E-14-8.250E+10)	0.838	NO
	Weighted median	17	1.152 (0.0517-25.7)	0.929	NO
	Simple mode	17	3.166 (0.0297-337)	0.635	NO
	Weighted mode	17	2.235 (0.0204-244)	0.741	NO
Tea intake	MR Egger	40	3.993 (0.3749-42.53)	0.258	NO
	Weighted median	40	1.857 (0.397-8.69)	0.432	NO
	Simple mode	40	0.349 (0.0108-11.26)	0.556	NO
	Weighted mode	40	1.452 (0.2356-8.95)	0.69	NO
Fresh fruit intake	MR Egger	53	1.1 (0.00624-193.93)	0.971	NO
	Weighted median	53	0.987 (0.10977-8.87)	0.99	NO
	Simple mode	53	9.456 (0.07536-1186.59)	0.366	NO
	Weighted mode	53	0.24 (0.00201-28.57)	0.561	NO
Cereal intake	MR Egger	39	1.05 (0.00494-224.87)	0.985	NO
	Weighted median	39	3.04 (0.52881-17.47)	0.213	NO
	Simple mode	39	3.42 (0.14099-83.07)	0.454	NO
	Weighted mode	39	4.64 (0.31902-67.62)	0.268	NO
Salad / raw vegetable intake	MR Egger	19	369.323 (0.002085-65400000)	0.351	NO
	Weighted median	19	0.159 (0.004693-5.4)	0.307	NO
	Simple mode	19	0.147 (0.00026-82.9)	0.56	NO
	Weighted mode	19	0.218 (0.000382-124)	0.643	NO
Coffee intake	MR Egger	38	3.203 (0.3772-27.19)	0.293	NO
	Weighted median	38	1.013 (0.1979-5.18)	0.988	NO
	Simple mode	38	1.423 (0.0502-40.38)	0.837	NO
	Weighted mode	38	2.662 (0.4316-16.42)	0.298	NO
Dried fruit intake	MR Egger	41	41.47 (0.132-13041.7)	0.212	NO
	Weighted median	41	4.1 (0.682-24.7)	0.123	NO
	Simple mode	41	9.65 (0.174-535.7)	0.275	NO
	Weighted mode	41	10.21 (0.284-366.8)	0.211	NO

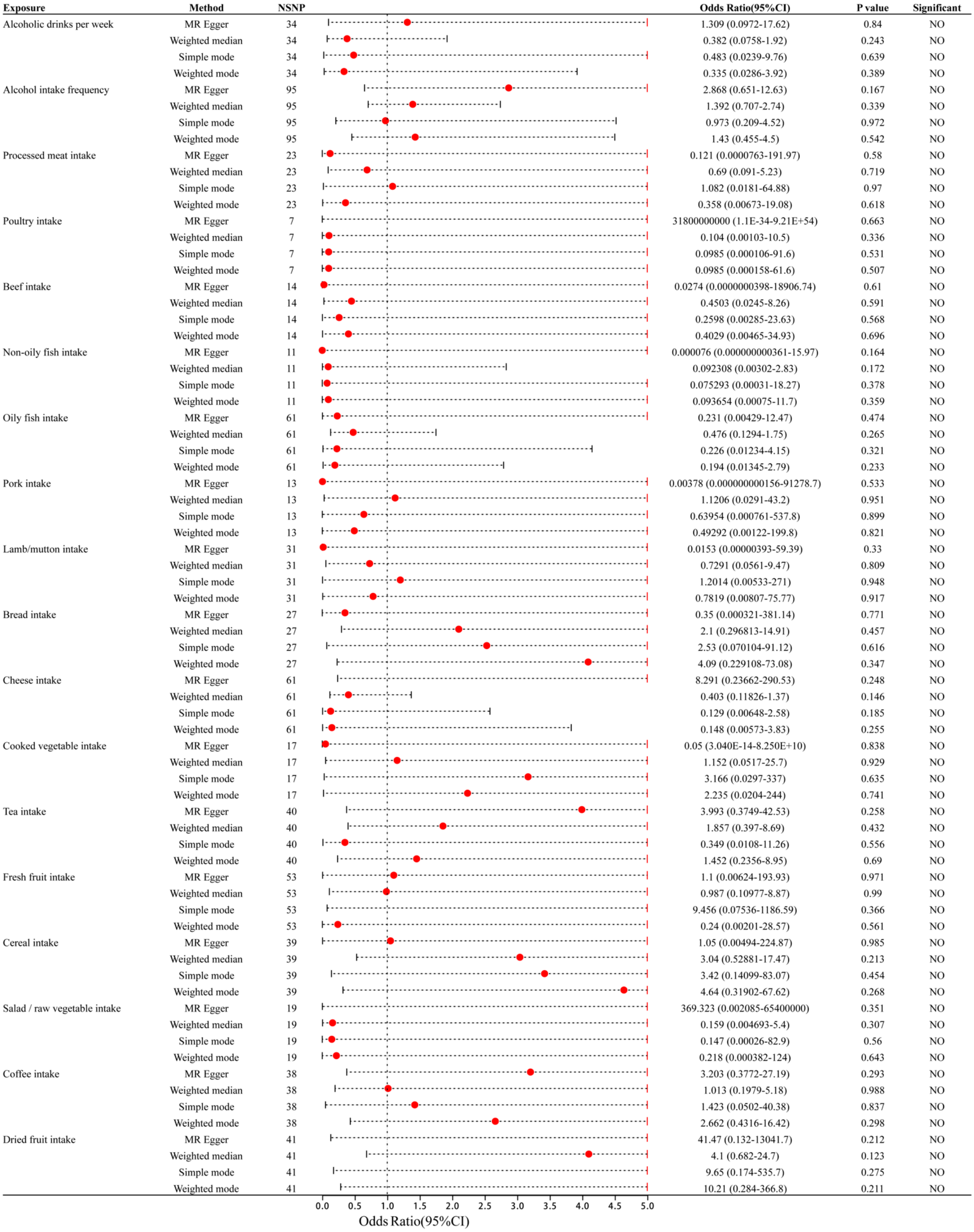

Figure S3: Leave-one-out plot for the effect of fresh fruit intake on intracranial aneurysms (ebi-a-GCST90018815).

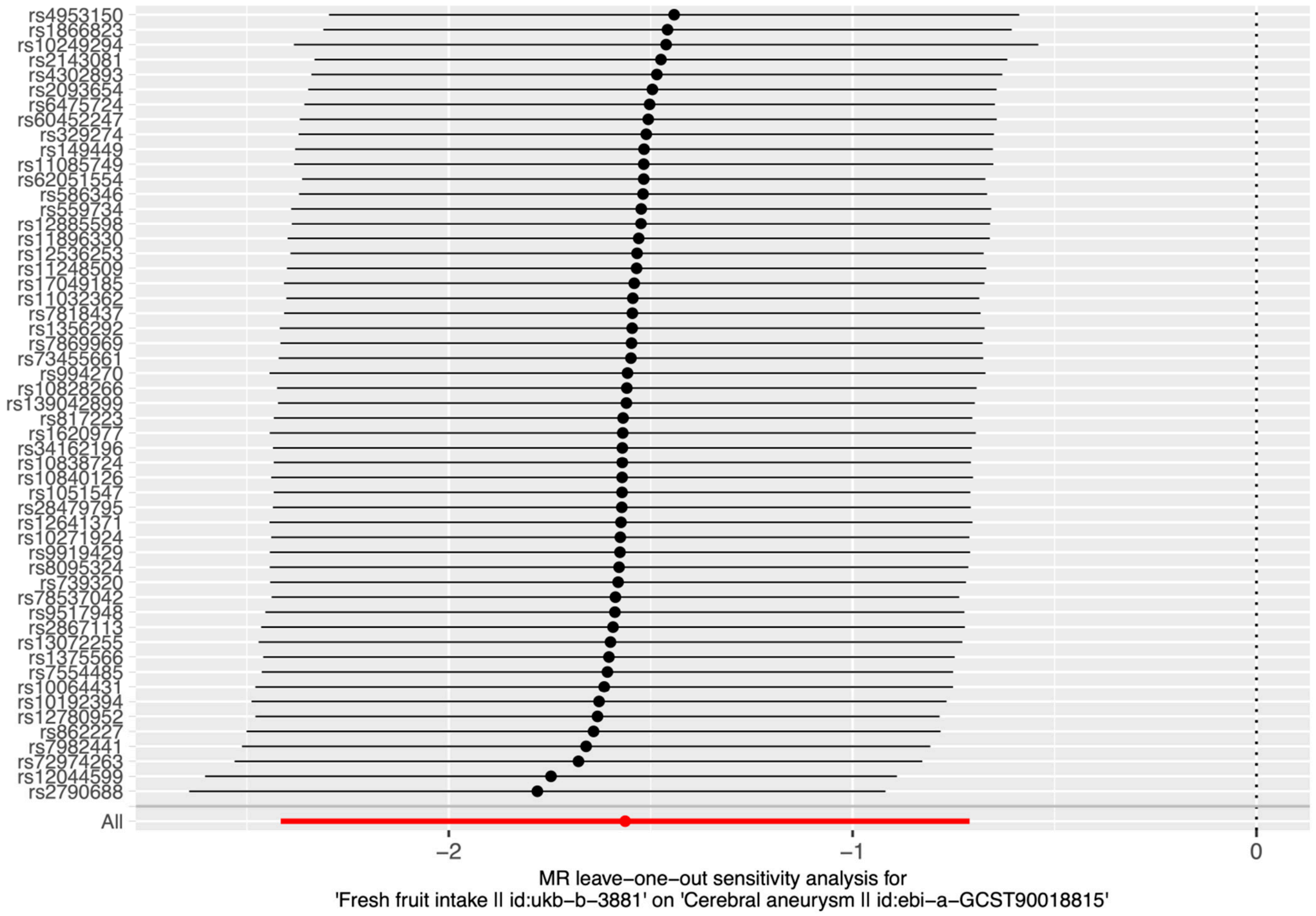

Figure S4: Leave-one-out plot for the effect of fresh fruit intake on intracranial aneurysms (finn-b-I9_ANEURYSM).

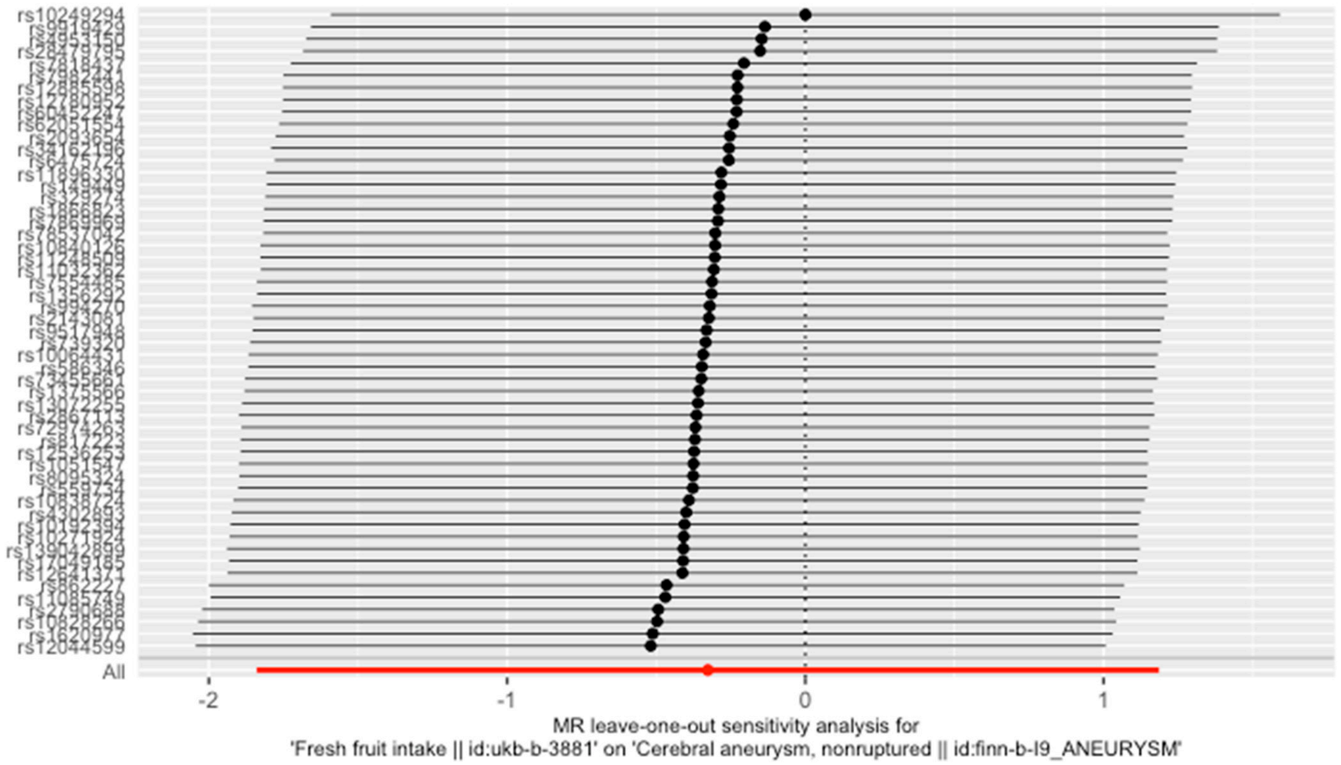

Figure S5: Leave-one-out plot for the effect of lamb/mutton intake on intracranial aneurysms (ebi-a-GCST90018815).

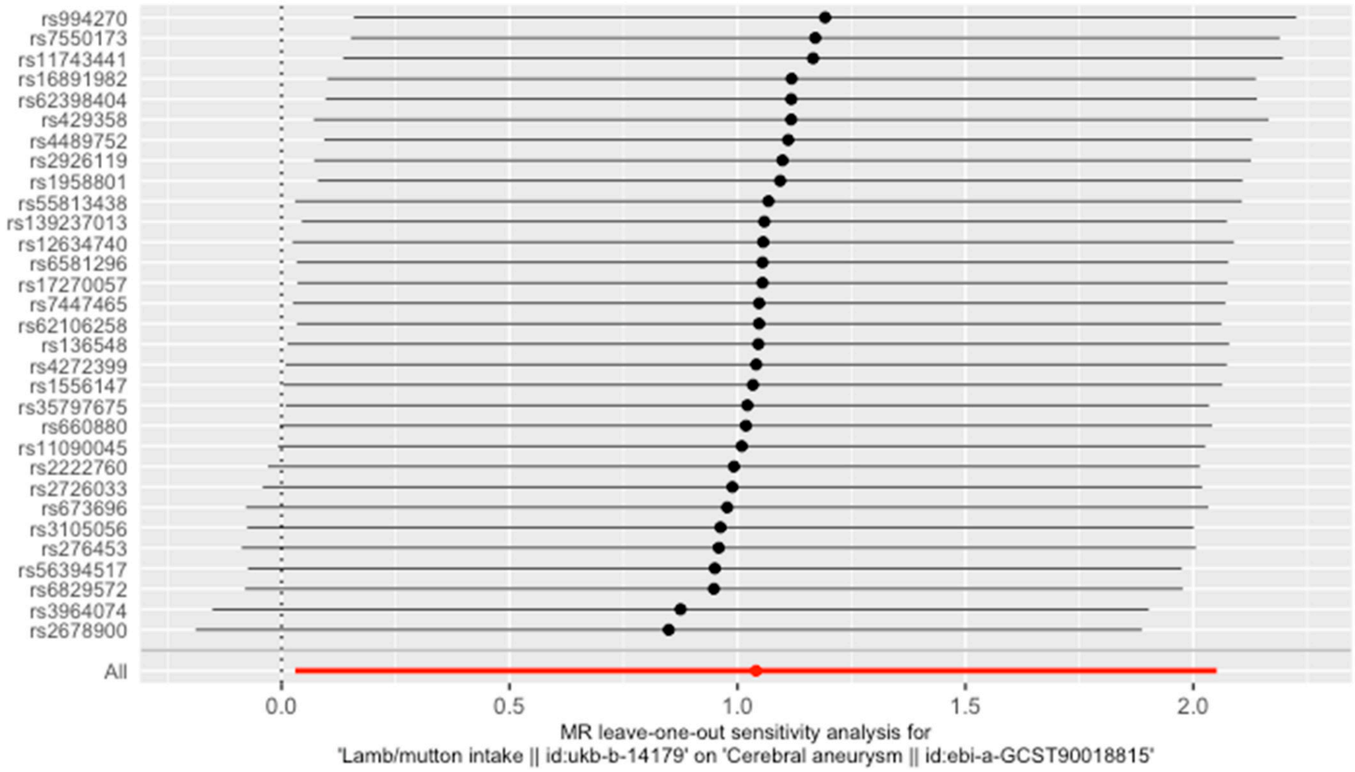

Figure S6: Leave-one-out plot for the effect of lamb/mutton intake on intracranial aneurysms (finn-b-I9_ANEURYSM).

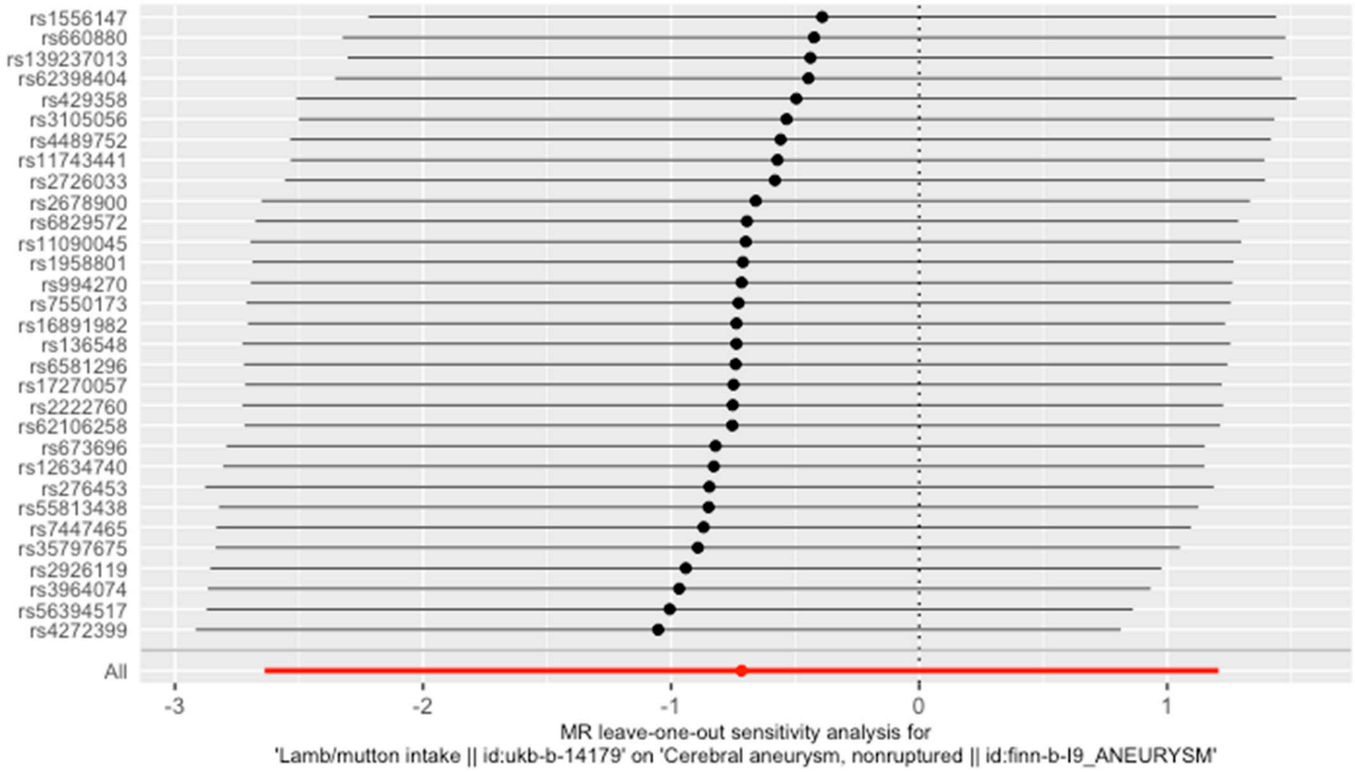

Figure S7: Scatter plot for the effect of fresh fruit intake on intracranial aneurysms (ebi-a-GCST90018815).

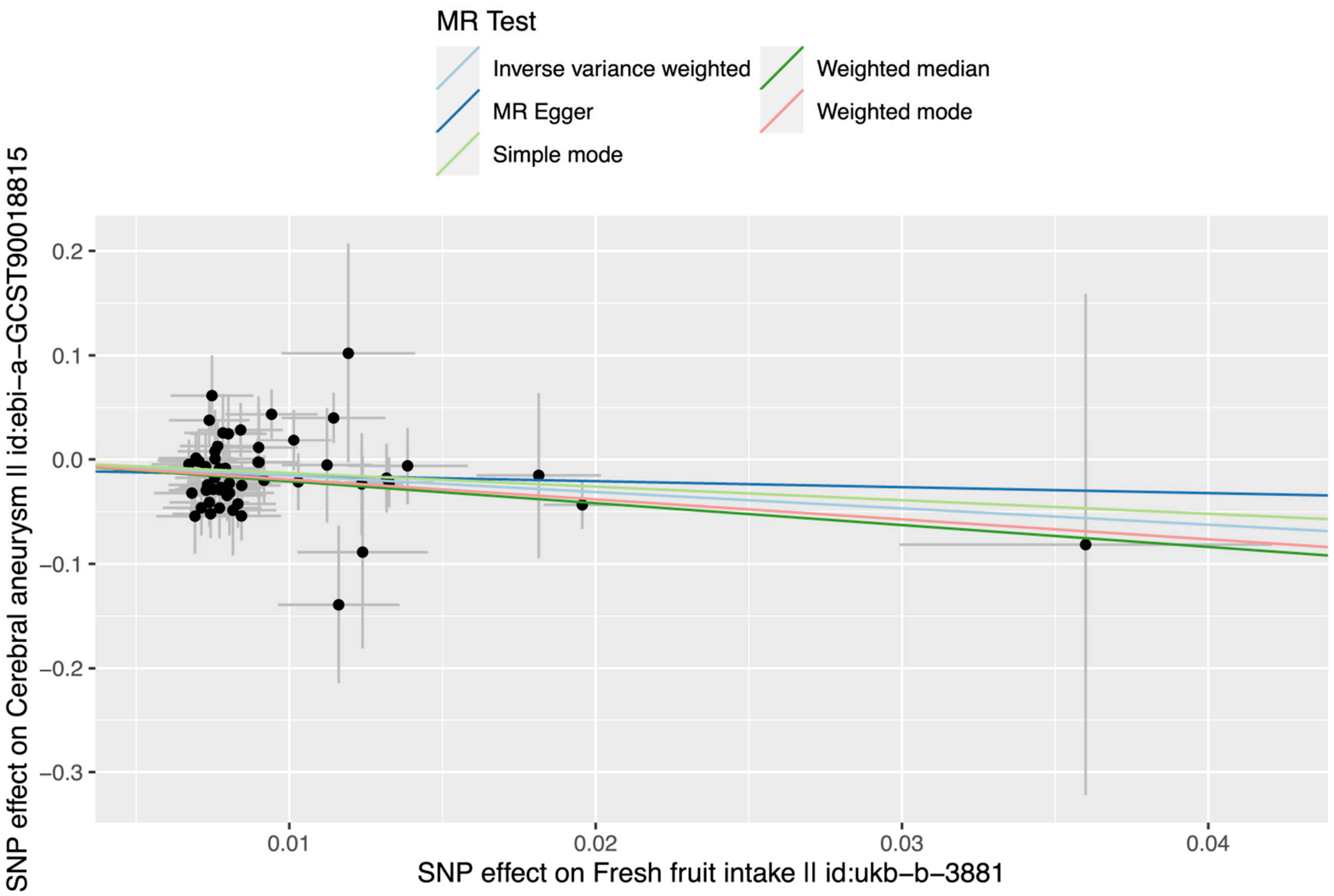

Figure S8: Scatter plot for the effect of fresh fruit intake on intracranial aneurysms (finn-b-I9_ANEURYSM).

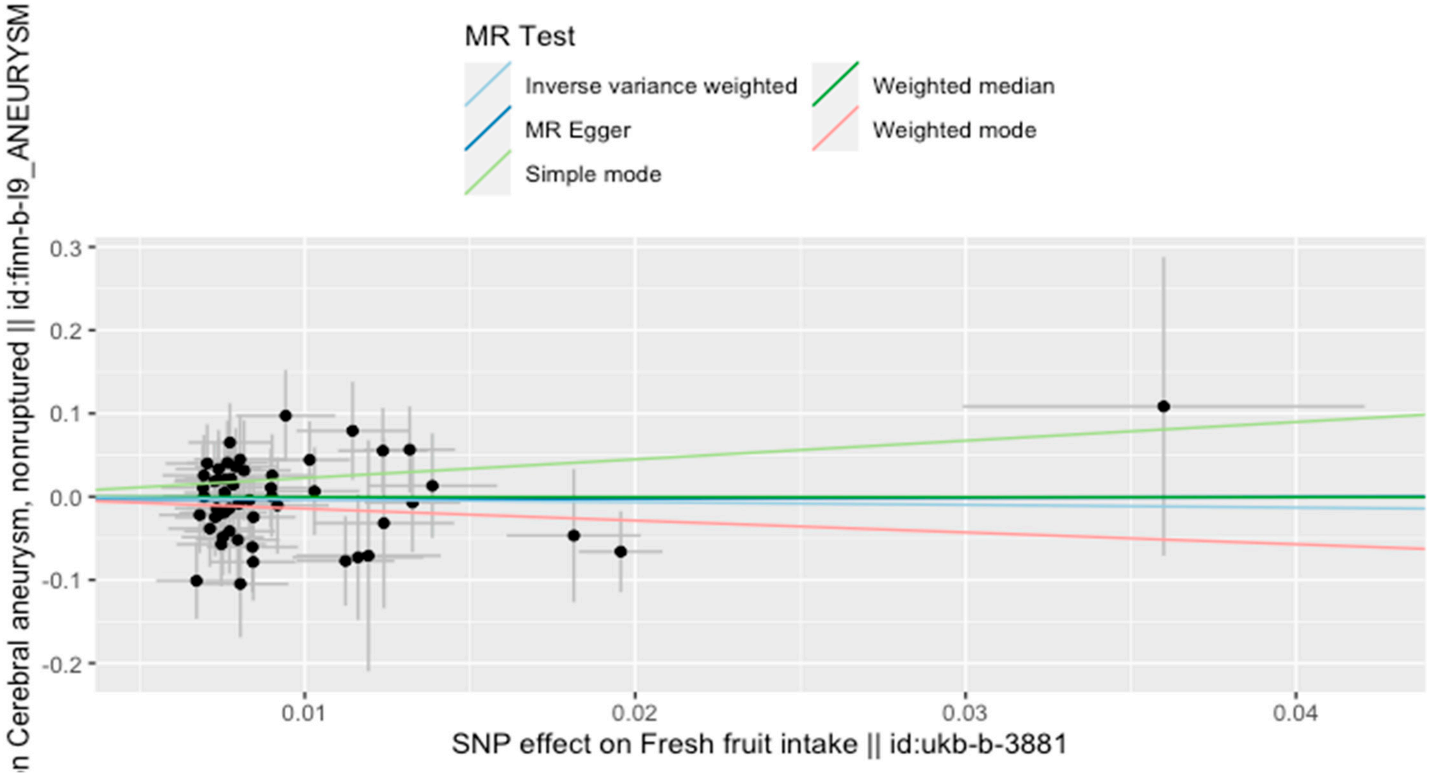

Figure S9: Scatter plot for the effect of lamb/mutton intake on intracranial aneurysms (ebi-a-GCST90018815).

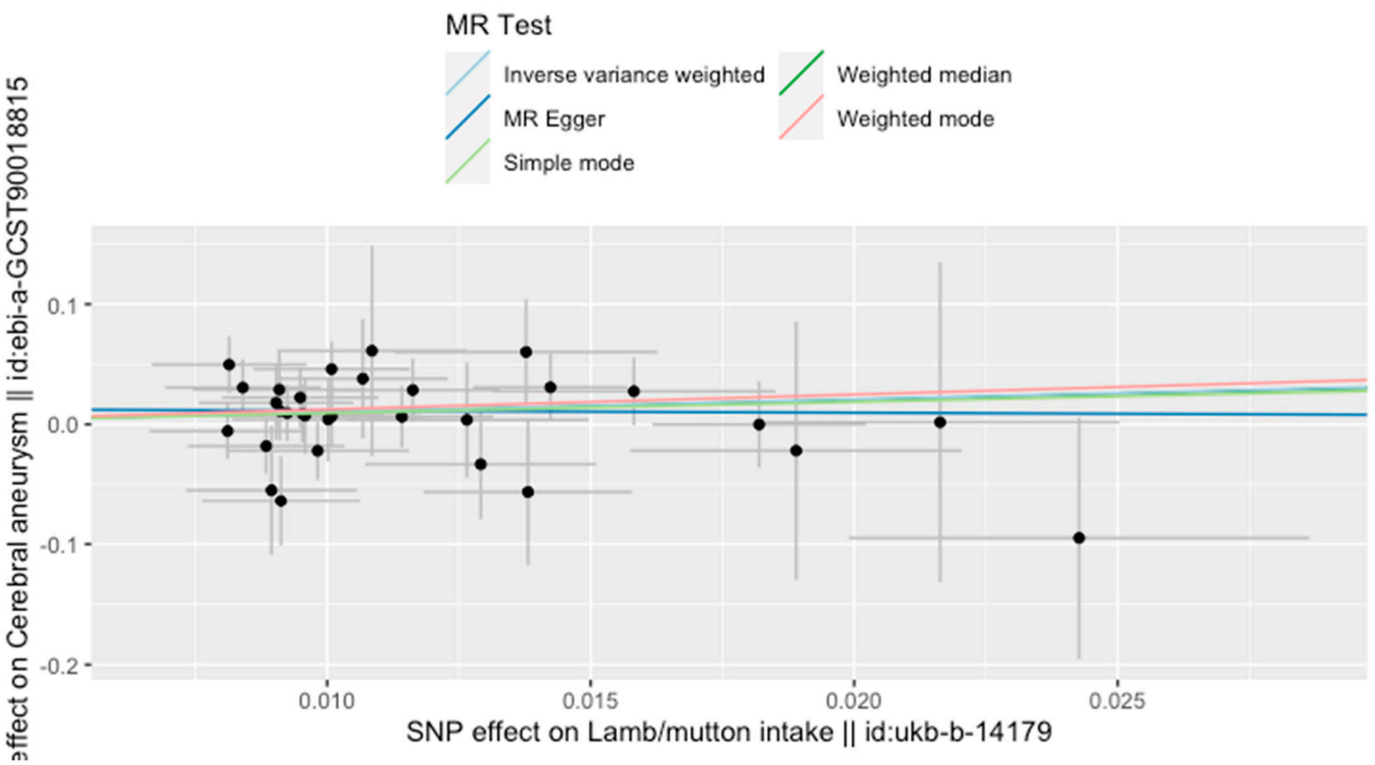

Figure S10: Scatter plot for the effect of lamb/mutton intake on intracranial aneurysms (finn-b-I9_ANEURYSM).

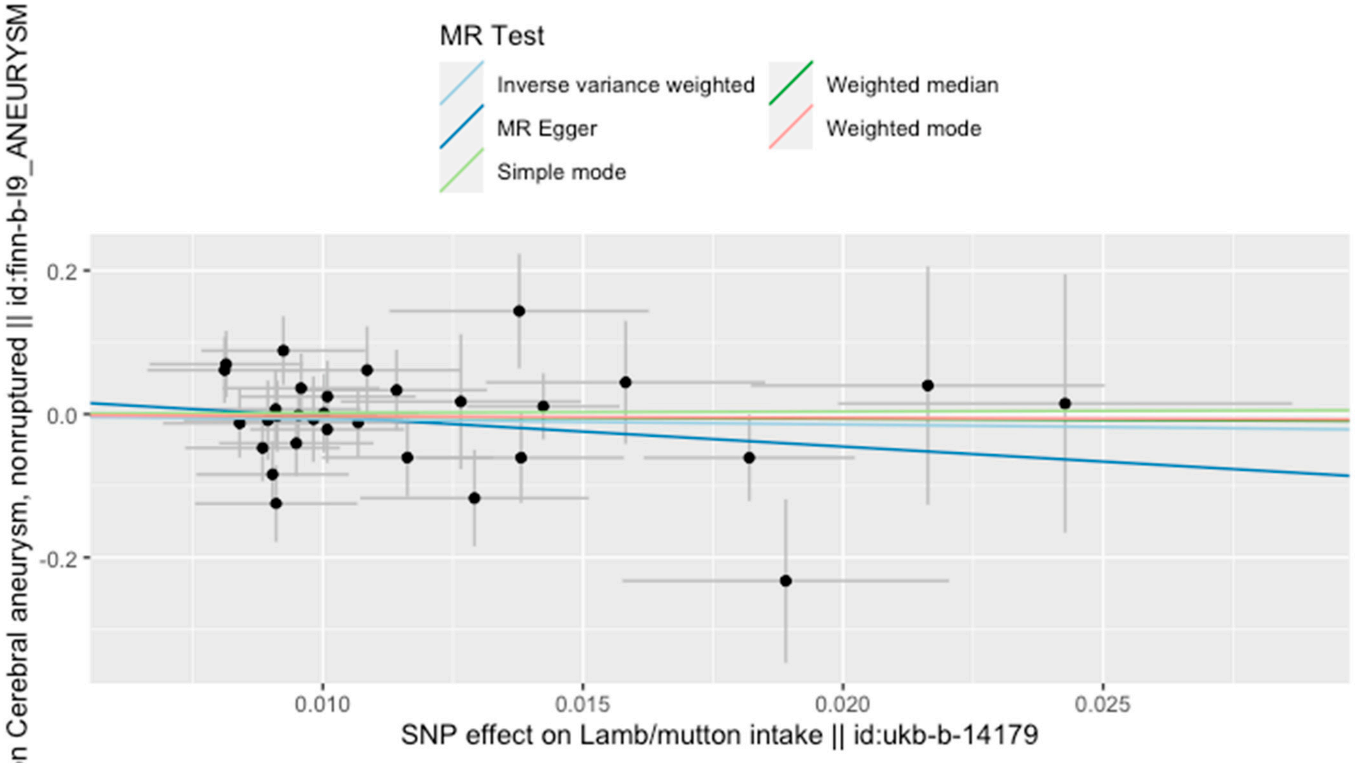

Figure S11: Funnel plot for the effect of fresh fruit intake on intracranial aneurysms (ebi-a-GCST90018815).

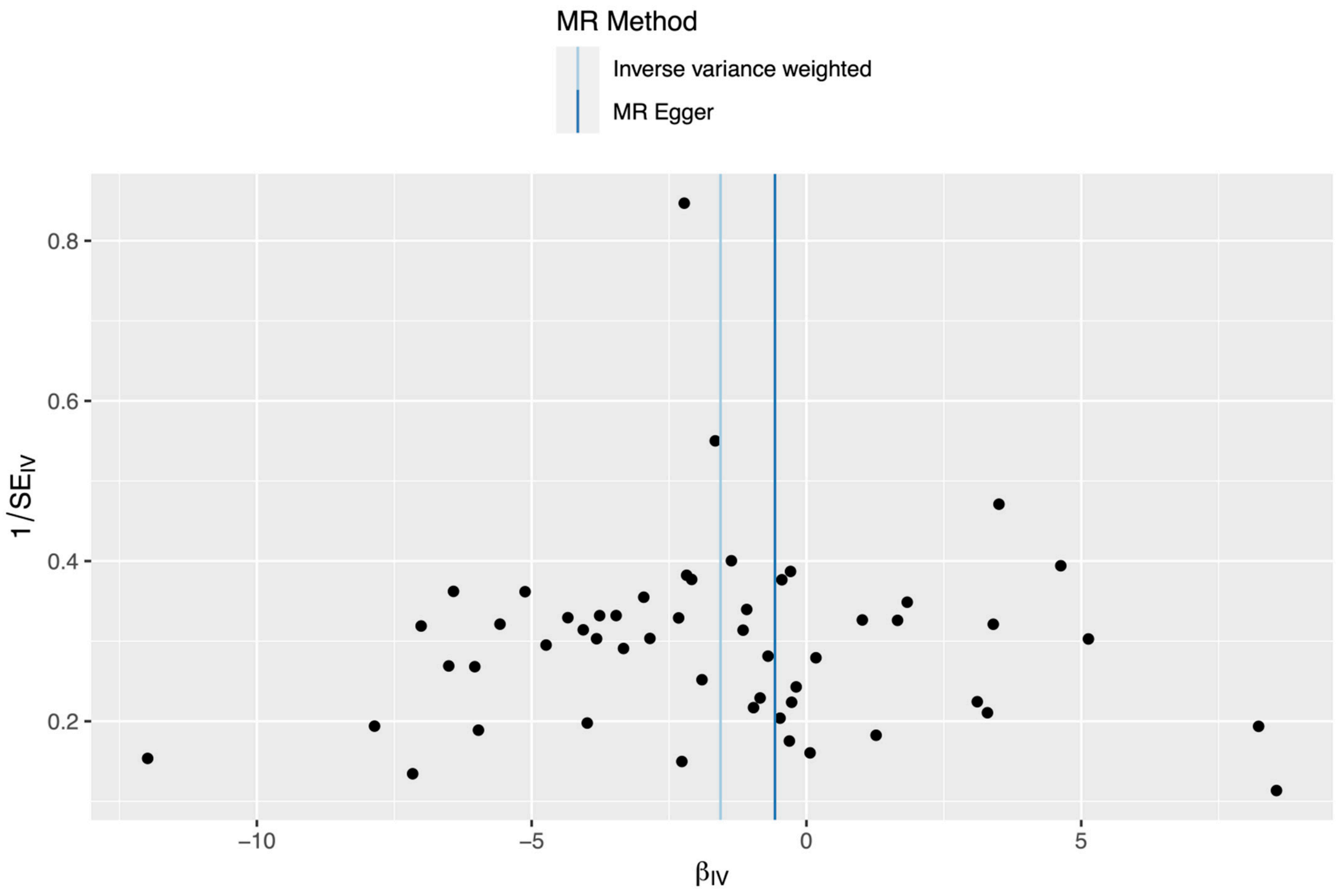

Figure S12: Funnel plot for the effect of fresh fruit intake on intracranial aneurysms (finn-b-I9_ANEURYSM).

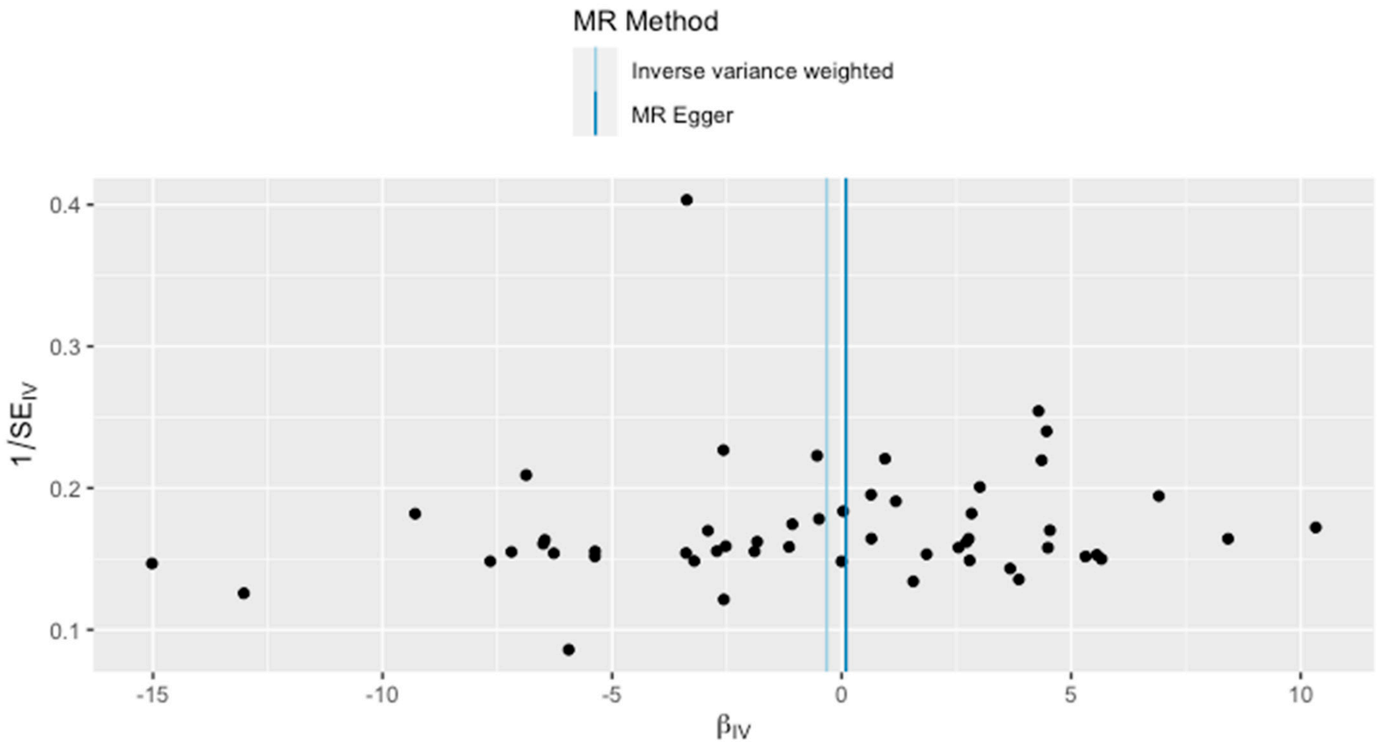

Figure S13: Funnel plot for the effect of lamb/mutton intake on intracranial aneurysms (ebi-a-GCST90018815).

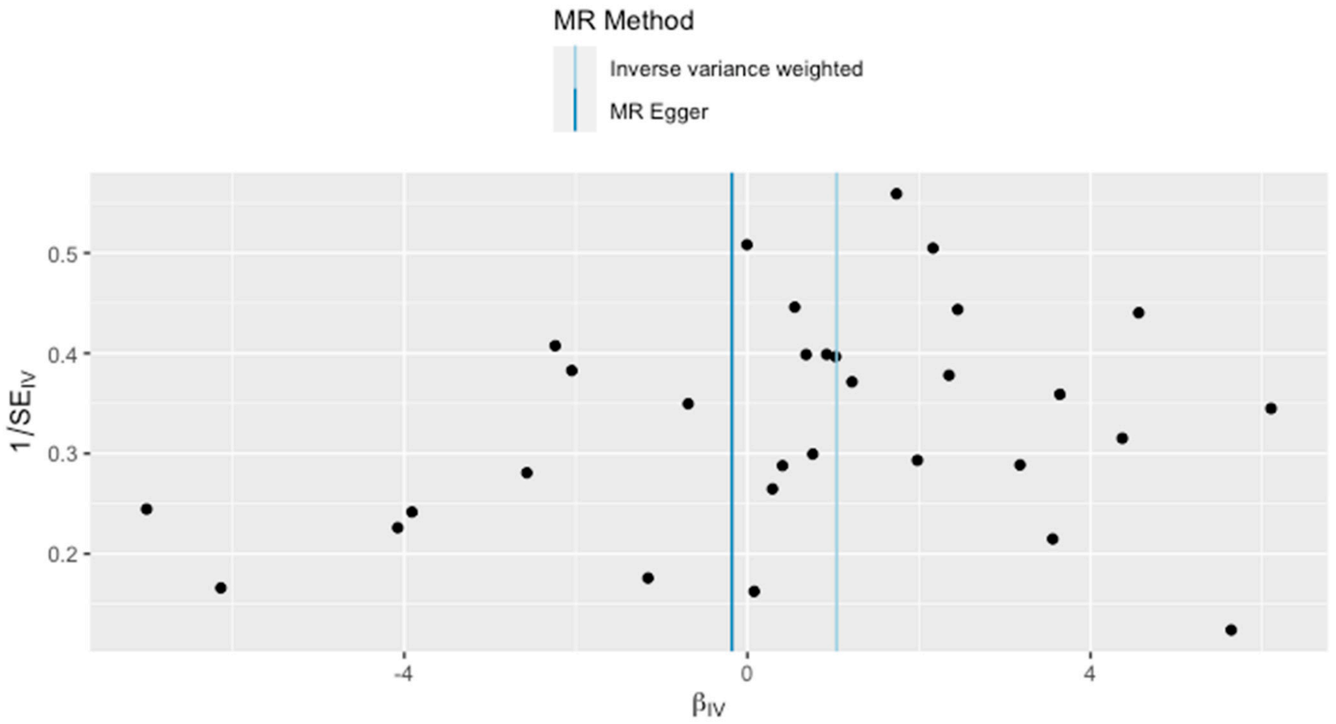

Figure S14: Funnel plot for the effect of lamb/mutton intake on intracranial aneurysms (finn-b-I9_ANEURYSM).

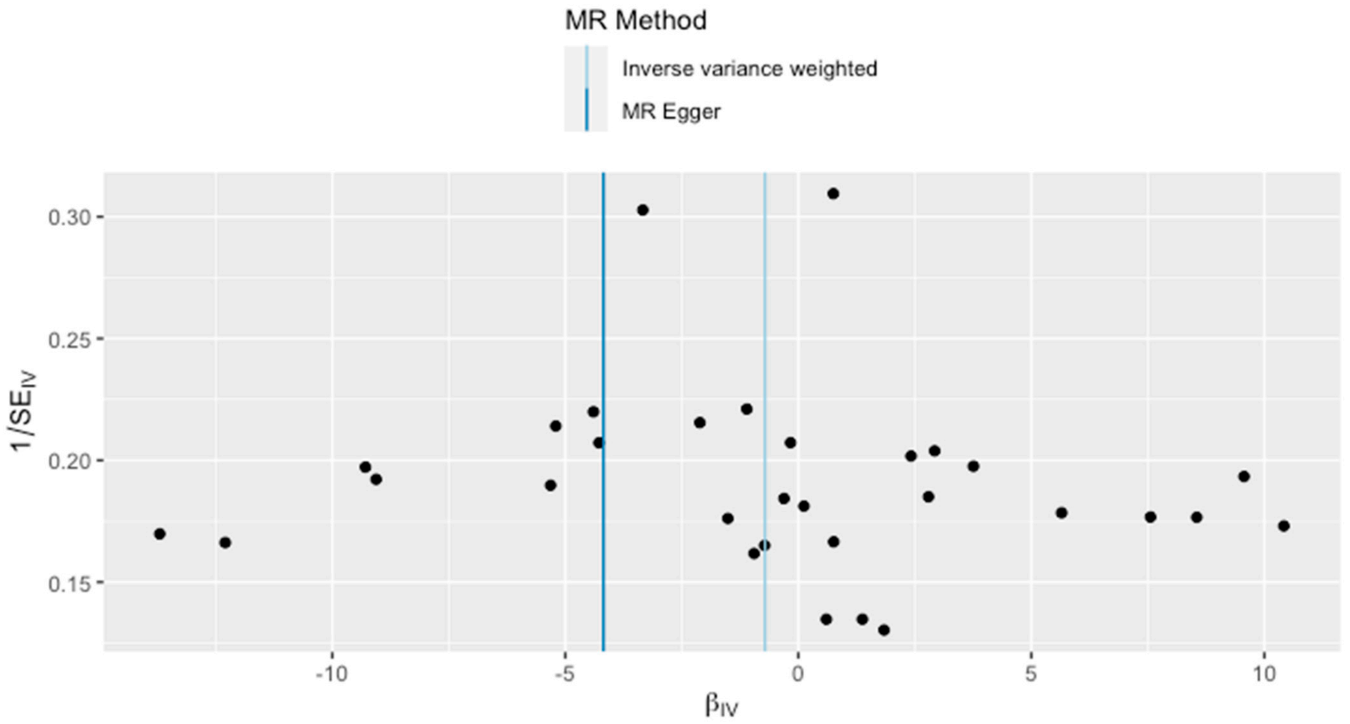

Figure S15: Forest plot for the effect of fresh fruit intake on intracranial aneurysms (ebi-a-GCST90018815).

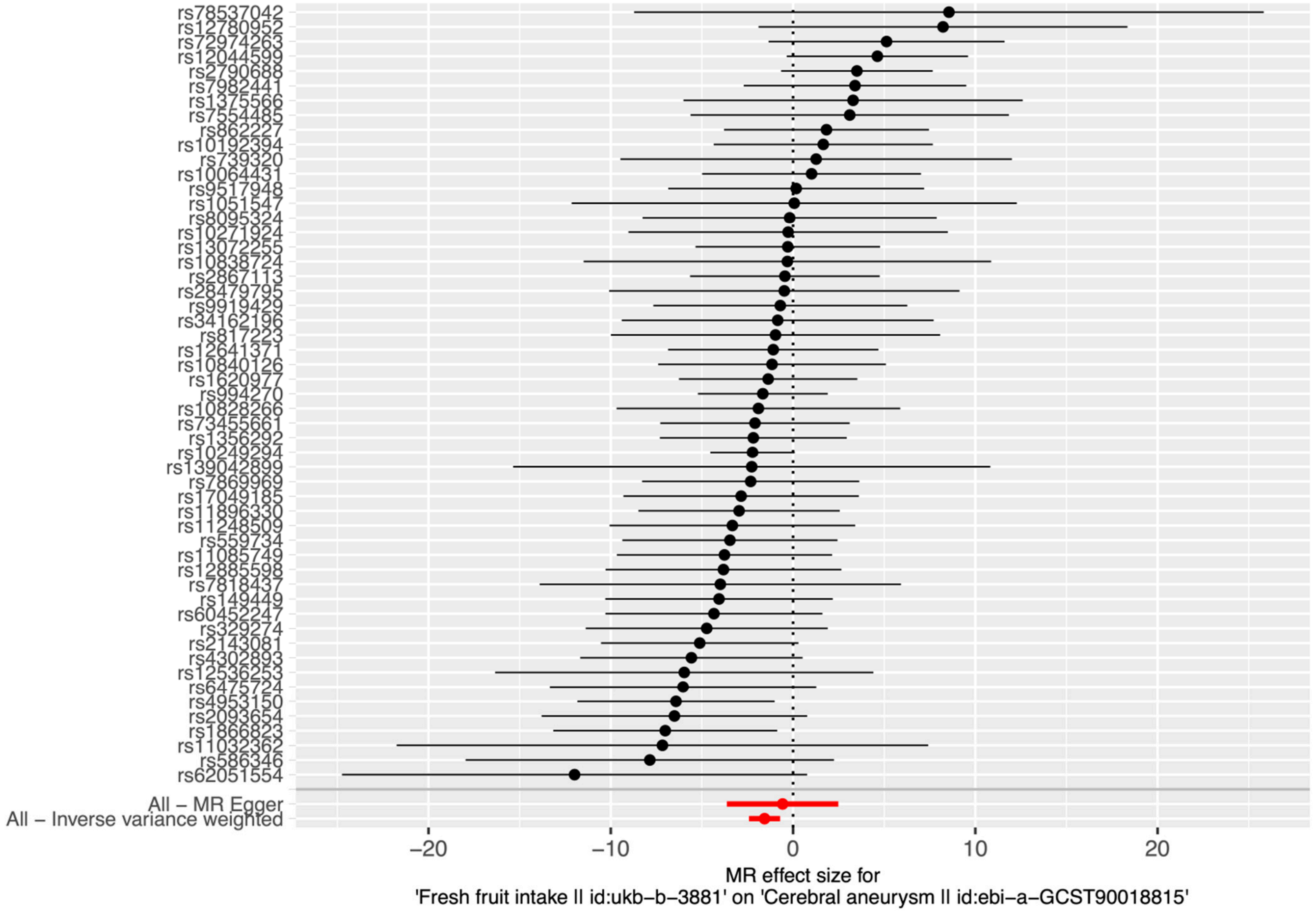

Figure S16: Forest plot for the effect of fresh fruit intake on intracranial aneurysms (finn-b-I9_ANEURYSM).

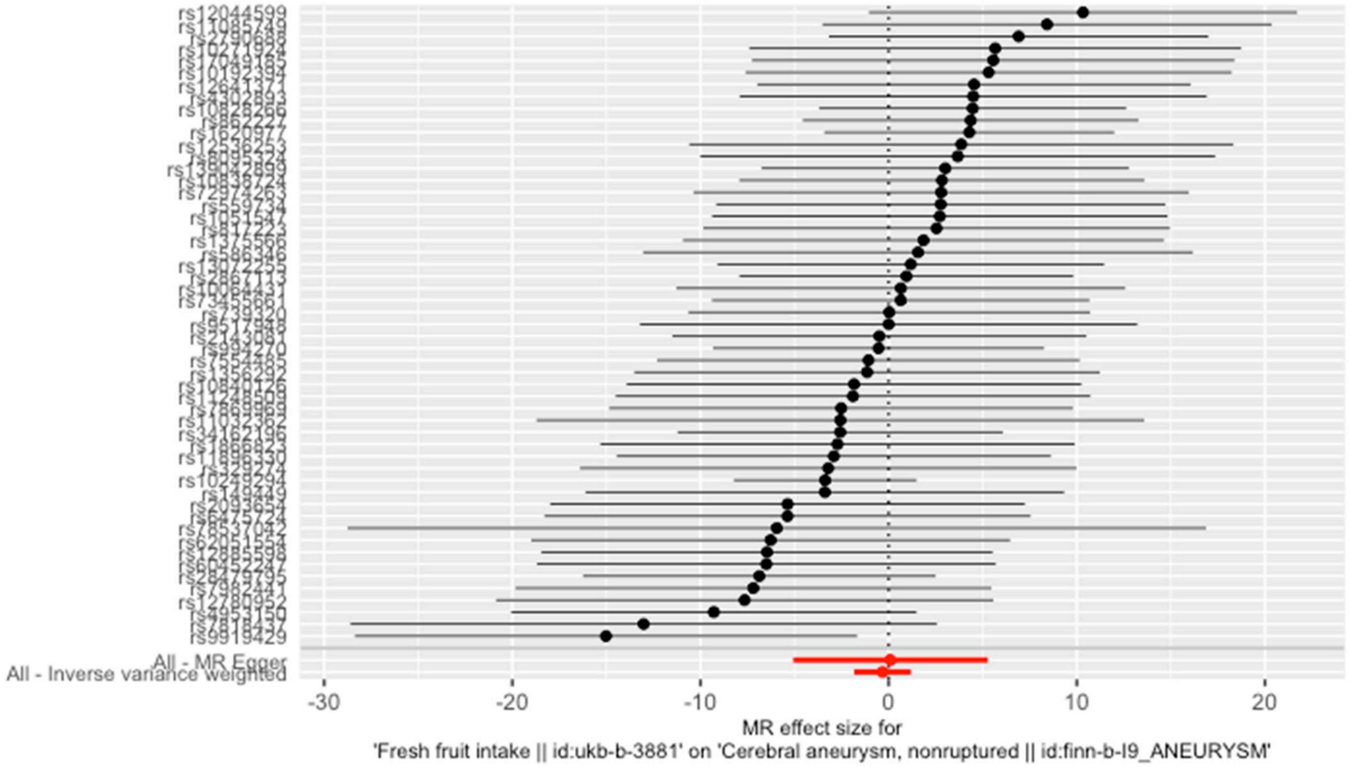

Figure S17: Forest plot for the effect of lamb/mutton intake on intracranial aneurysms (ebi-a-GCST90018815).

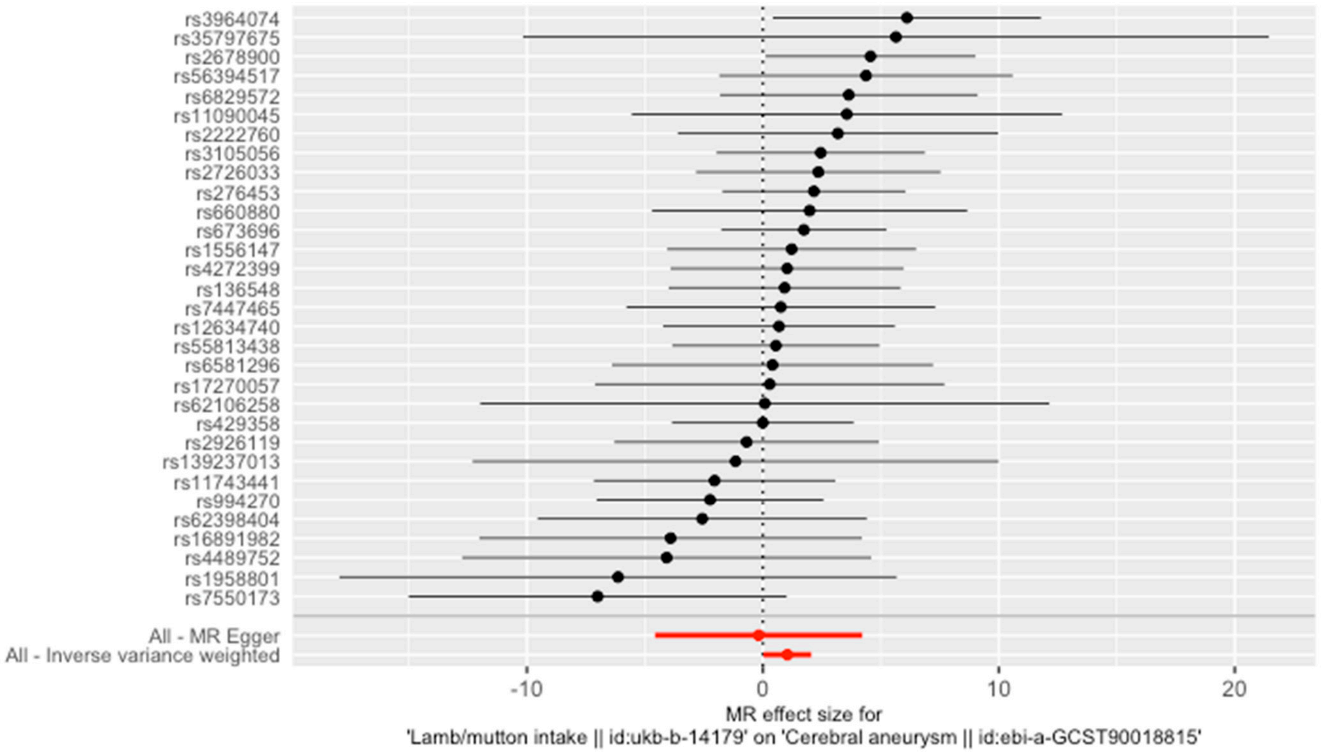

Figure S18: Forest plot for the effect of lamb/mutton intake on intracranial aneurysms (finn-b-I9_ANEURYSM).

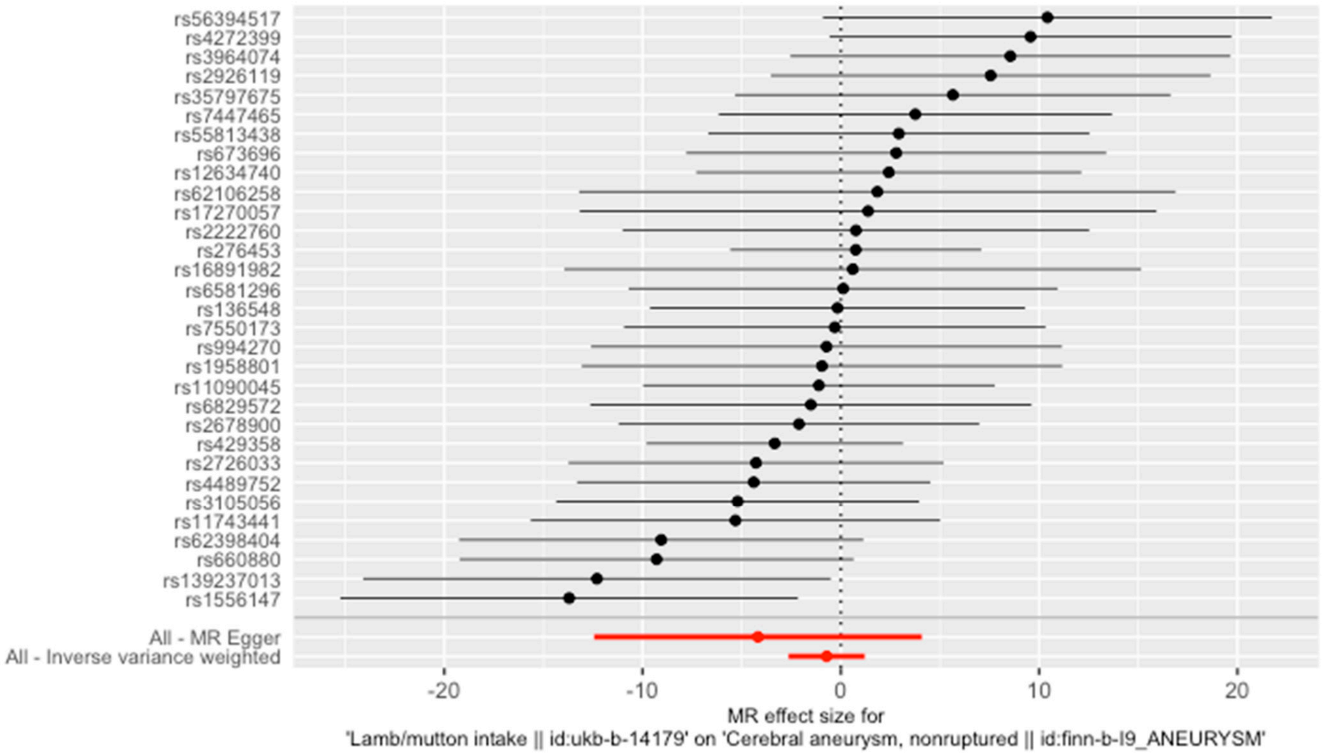

Supplement: Supplementary file 1 [file biomedicines-13-00533-s001.zip › biomedicines-3409192-supplementary.pdf]
